# Phytochemical Characterization, Anti-Oxidant, Anti-Enzymatic and Cytotoxic Effects of *Artemisia verlotiorum* Lamotte Extracts: A New Source of Bioactive Agents

**DOI:** 10.3390/molecules27185886

**Published:** 2022-09-10

**Authors:** Shanoo Suroowan, Eulogio Jose Llorent-Martínez, Gokhan Zengin, Stefano Dall’Acqua, Stefania Sut, Kalaivani Buskaran, Sharida Fakurazi, Mohamad Fawzi Mahomoodally

**Affiliations:** 1 Department of Health Sciences, Faculty of Medicine and Health Sciences, University of Mauritius, Réduit, Moka 80837, Mauritius; 2 Department of Physical and Analytical Chemistry, University of Jaén, Campus Las Lagunillas S/N, E-23071 Jaén, Spain; 3 Department of Biology, Science Faculty, Selcuk University, Konya 42130, Turkey; 4 Department of Pharmaceutical and Pharmacological Sciences, University of Padova, Via Marzolo 5, 35131 Padova, Italy; 5 Laboratory of Natural Medicine and Product Research, Institute of Bioscience, Universiti Putra Malaysia (UPM), Serdang 43400, Selangor Darul Ehsan, Malaysia; 6 Department of Human Anatomy, Faculty of Medicine & Health Sciences, Universiti Putra Malaysia (UPM), Serdang 43400, Selangor Darul Ehsan, Malaysia; 7 Center for Transdisciplinary Research, Department of Pharmacology, Saveetha Dental College, Saveetha Institute of Medical and Technical Science, Chennai 600077, India; 8 Centre of Excellence for Pharmaceutical Sciences (Pharmacen), North West University, Potchefstroom 2520, South Africa

**Keywords:** *Artemisia verlotiorum*, cytotoxicity, amylase, tyrosinase, phyto-pharmaceutics

## Abstract

*Artemisia verlotiorum* Lamotte is recognized medicinally given its long-standing ethnopharmacological uses in different parts of the world. Nonetheless, the pharmacological properties of the leaves of the plant have been poorly studied by the scientific community. Hence, this study aimed to decipher the phytochemicals; quantify through HPLC-ESI-MS analysis the plant’s biosynthesis; and evaluate the antioxidant, anti-tyrosinase, amylase, glucosidase, cholinesterase, and cytotoxicity potential on normal (NIH 3T3) and human liver and human colon cancer (HepG2 and HT 29) cell lines of this plant species. The aqueous extract contained the highest content of phenolics and phenolic acid, methanol extracted the most flavonoid, and the most flavonol was extracted by ethyl acetate. The one-way ANOVA results demonstrated that all results obtained were statistically significant at *p* < 0.05. A total of 25 phytoconstituents were identified from the different extracts, with phenolic acids and flavonoids being the main metabolites. The highest antioxidant potential was recorded for the aqueous extract. The best anti-tyrosinase extract was the methanolic extract. The ethyl acetate extract of *A. verlotiorum* had the highest flavonol content and hence was most active against the cholinesterase enzymes. The ethyl acetate extract was the best α-glucosidase and α-amylase inhibitor. The samples of *Artemisia verlotiorum* Lamotte in both aqueous and methanolic extracts were found to be non-toxic after 48 h against NIH 3T3 cells. In HepG2 cells, the methanolic extract was nontoxic up to 125 µg/mL, and an IC_50_ value of 722.39 µg/mL was recorded. The IC_50_ value exhibited in methanolic extraction of *A. verlotiorum* was 792.91 µg/mL in HT29 cells. Methanolic extraction is capable of inducing cell cytotoxicity in human hepatocellular carcinoma without damaging normal cells. Hence, *A. verlotiorum* can be recommended for further evaluation of its phytochemical and medicinal properties.

## 1. Introduction

The therapeutic properties and safety margin of medicinal plants are known due to their extensive use in traditional systems of medicine since antiquity [1]. Despite the existence of diverse conventional medicinal agents, the versatility of medicinal plants in addressing various targets and their low side effects make them valuable therapeutic candidates [2]. In addition, the increase in the complexity of maladies, emergence of new infections, and development of resistance among various pathogens to available conventional medications has directed researchers to place their trust in medicinal plant species for the development of potential novel drugs once again [3].

Over 500,000 plant species are present worldwide [4]. Plant species with a diligent history of ethnomedicinal use are expected to hold therapeutic components [3]. In fact, the longstanding ethnomedicinal use of a plant species can be correlated with the presence of bioactive compounds within. Indeed, this is a common methodology adopted by researchers in an attempt to decipher novel therapeutic molecules from plant species [5,6].

In some instances, despite their long-standing use in traditional medicine, the pharmacological properties of several plant species are not sufficiently researched and hence not documented [7]. Similarly, *Artemisia verlotiorum* Lamotte has been employed as a traditional remedy in various parts of the world, such as in Brazil, Italy, and Mauritius among other countries, but limited research has been conducted on this species to investigate its biochemical profile [8,9,10]. It belongs to the Asteraceae family, which comprises about 500 herbal species.

The traditional uses associated with *A. verlotiorum* have been recorded mainly against hypertension, fever, influenza, psoriasis, and circulatory, digestive, genito-urinary, renal failure, and respiratory disorders [8,9,10,11]. To establish the medicinal properties of a plant species, it remains important to generate evidence through the determination of its phytochemistry and study of its pharmacological properties [12].

Plant species consist of a myriad of metabolites that can sometimes be challenging to separate and identify. Liquid chromatography–mass spectrometry (LC-MS) is a technique that has been used for over 40 years to answer this challenge. Over time, it has been further developed, and high-performance liquid chromatography is now a one-stop problem solver for phytochemical separation challenges [13].

The interest in isolating and distinctly identifying the panoply of secondary metabolites in plant species is only one side of the coin. The other side focuses on elucidating the mysteries behind the biological properties of the plant species—most importantly, for humans. They present an interesting source of compounds to be evaluated for their medicinal properties. Hence, a first-line approach in such an evaluation is the use of different assays to investigate their biological properties in vitro [14].

This study aims to document the phytochemical profile of *A. verlotiorum* through in-vitro-based assays and the HPLC-ESI-MS^n^ method and to study its pharmacological properties, such as its antioxidant, antidiabetic, anticancer, antityrosinase, and metal chelating properties, and its potential against neurodegenerative disorders such as Alzheimer’s disease in vitro.

## 2. Results

### 2.1. Phytochemical Profile

The aqueous extract had the highest concentration of phenolic content (104.42 mg GAE/g extract) and total phenolics (59.26 mg CE/g extract), followed by the methanolic (56.5 mg GAE/g extract & 32.38 mg CE/g extract) and ethyl acetate extracts, respectively (25.28 mg GAE/g extract and no phenolic acid detected). The total flavonoid content of the methanolic extract was highest (31.38 mg RE/g extract), ahead of the ethyl acetate (13.81 mg RE/g extract) and aqueous extract (10.7 mg RE/g extract), while the highest flavonol content (2.23 mg CAE/g extract) was recorded for the ethyl acetate extract, followed by the aqueous (1.63 mg CAE/g extract) and methanolic extract (1.44 mg CAE/g extract). The one-way ANOVA results demonstrated that all results retrieved were statistically significant at *p* < 0.05. The results are shown in Table 1.

### 2.2. HPLC-ESI-MS^n^ Analysis

Following identification of the phytochemicals from the extracts, it was important to further characterize them; hence, HPLC-ESI-MS^n^ analysis was conducted.

**Figure 1:**
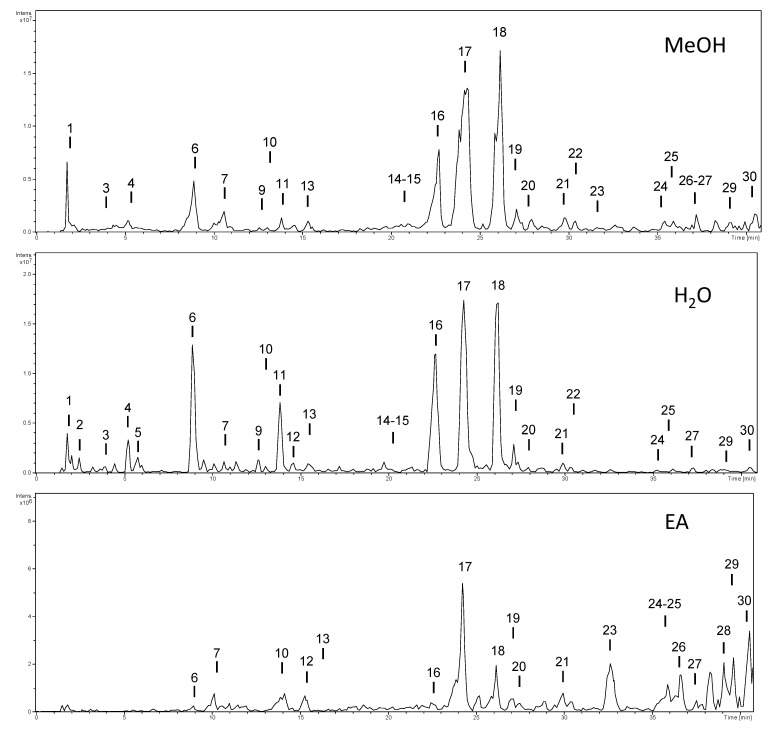
Base peak chromatograms of the extracts of *Artemisia verlotiorum*.

The characterization of the phytochemicals was carried out by HPLC-ESI-MS^n^ using the negative ion mode. Identification was performed using analytical standards—citric acid, chlorogenic acid, quercetin, rutin, vicenin-2—as well as bibliographic information. The base peak chromatograms of the extracts are shown in Figure 1, and the characterization is shown in Table 2. Compounds were numbered according to their elution order, keeping the same numbering in all extracts.

Compound **1** was identified as citric acid. Compound **2** was characterized as quinic acid due to the base peak at *m*/*z* 173, and it was differentiated from citric acid (both compounds have the deprotonated molecular ion at *m*/*z* 191) due to the identification of citric acid with an analytical standard.

Compound **3** suffered the neutral loss of 162 Da (hexoside) to yield the MS^2^ base peak at *m*/*z* 153, whose fragmentation pattern was consistent with dihydroxybenzoic acid (an analytical standard of protocatechuic acid was used to compare the fragmentation pattern).

Compounds **5** and **8** were characterized as caffeoylglucaric and dicaffeoylglucaric acids based on bibliographic information [15].

Compound **7**, [M − H]^−^ at *m*/*z* 387, was characterized as the lignan medioresinol due to the fragment ions at *m*/*z* 207 and 163, whereas compound **19**, with additional 162 Da, was characterized as a hexoside [16].

Compounds **10** and **12** were identified as 5-*p*-coumaroylquinic acid and 5-feruloylquinic acid based on the hierarchical scheme proposed by Clifford et al. [17].

Compounds **11**, **16**, **17**, **18**, and **22** were characterized as dicaffeoylquinic acid isomers due to the deprotonated molecular ion at *m*/*z* 515 and MS^2^ base peak at *m*/*z* 353. These compounds were the main ones in all the analyzed extracts. Compound **27**, with additional 162 Da, was characterized as a tricaffeoylquinic acid.

Compounds **15** and **20** displayed neutral losses of hexoside moieties (162 Da) to yield quercetin at *m*/*z* 301 (identified by comparison with an analytical standard), so they were identified as quercetin-*O*-hexoside and quercetin-*O*-hexoside-*O*-hexoside.

Compound **21**, with deprotonated molecular ion at *m*/*z* 529, exhibited fragment ions at *m*/*z* 367, 353, and 191. These fragments were consistent with feruloyl-caffeoyl-quinic acid [17].

Compound **23** presented the deprotonated molecular ion at *m*/*z* 287, and its fragmentation pattern was consistent with the flavanone eriodictyol [18]. Compound **25** was identified as luteolin by comparison with an analytical standard.

Compounds **29** and **30** were characterized as the oxylipins oxo-dihydroxy-octadecenoic acid and trihydroxy-octadecenoic acid based on bibliographic information [19].

### 2.3. Quantification of Phytochemicals

First, the most abundant compounds were quantified by UV using analytical standards of neochlorogenic acid, chlorogenic acid, and quercetin. Chlorogenic acid was used to quantify dicaffeoylquinic acids. Analytical signals were recorded at 320 nm for phenolic acids and 350 nm for the flavonoid. Results are shown in Table 3. It can be observed that the aqueous extract presented the highest concentration of phenolics, followed by methanol and ethyl acetate extracts, which is in line with the results obtained from the colorimetric methods above. Both methanol (76 mg g^−1^ DE) and aqueous (105 mg g^−1^ DE) extracts presented a significant concentration of phenolic compounds. The most abundant compounds in both aqueous and methanol extracts were compound **6** (chlorogenic acid) and compounds **16**, **17**, and **18** (dicaffeoylquinic acids). However, as can be seen in Table 4, only a few compounds could be quantified (the other compounds had very low UV signals). We thus also calculated the relative contribution of all compounds using the method of area normalization (Figure 1). Peak areas of each compound were obtained using the precursor ion, [M − H]^−^, (extracted ion chromatograms). Then, the relative contribution (in percentage) of each compound was calculated, and the heat map (the darker the color, the higher the abundance) was constructed (Table 4). Dicaffeoylquinic acids (mainly compound **17**) were the most abundant, representing approximately 75% of the total compounds in methanol and aqueous extracts, and 40% in the ethyl acetate extract. Therefore, dicaffeoylquinic acids and chlorogenic acids are the main contributors to the bioactivity observed in the other assays. The presence of these compounds in *Artemisia* species and their bioactivity have been previously reported [20,21].

### 2.4. Antioxidant Activity

The best free radical scavenger as demonstrated by the results of the DPPH, ABTS, FRAP, and CUPRAC methods was the aqueous extract (370.26, 669.46, 565.77, and 875.90 mg TE/g, respectively). The aqueous extract was followed by the methanolic and ethyl acetate extract in terms of antioxidant potential. The aqueous extract was also the best metal chelator and displayed the best activity in the phosphomolybdenum assay (Table 5).

### 2.5. Anti-Tyrosinase, Anti-Cholinesterase, and Anti-Glucosidase Activities

The best anti-tyrosinase activity was recorded for the methanolic extract (126.99 mgKAE/g), followed closely by the ethyl acetate extract (122.14 mgKAE/g), and finally the aqueous extract (53.26 mgKAE/g) (Table 6).

In this study, *A. verlotiorum* aqueous extract was not active against neither AChE nor BChE. The ethyl acetate extract of *A. verlotiorum* was most active against the cholinesterase enzymes (5.48 and 6.83 mgGALAE/g against AChE and BChE, respectively) followed by the *A. verlotiorum* methanolic extract (3.28 and 2.93 mgGALAE/g against AChE and BChE, respectively). *A. verlotiorum* ethyl acetate extract was the best α-glucosidase inhibitor, followed by the methanolic and aqueous extracts (1.71, 0.49, 0.31 mmolACAE/g, respectively).

### 2.6. Cytotoxicity Studies on Normal (NIH 3T3) and Cancer (HepG2 and HT 29) Cell Lines

In this study, the cytotoxicity potential of *A. verlotiorum* was investigated on normal human fibroblast (3T3), human hepatocellular carcinoma cells (HepG2), and human colorectal carcinoma cells (HT29).

**Figure 2:**
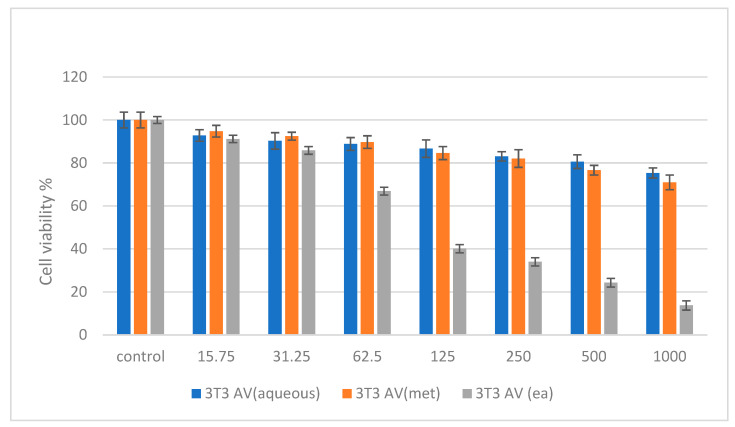
Cytotoxicity assay of *Artemisia verlotiorum* in aqueous, methanolic, and ethyl acetate extracts against normal 3T3 cells at 48 h.

Cytotoxicity studies were conducted on normal (NIH 3T3) and cancer (HepG2 and HT 29) cell lines by treating them with *A. verlotiorum* in aqueous, methanolic, and ethyl acetate extracts. Various concentrations of the samples were incubated for a maximum of 48 h. Figure 2 shows the percentage cell viability of the 3T3 cells after 48 h incubation for all the samples. The samples of *A. verlotiorum* Lamotte in both aqueous and methanolic extracts were found to be non-toxic after 48 h incubation. On the other hand, the ethyl acetate extract was nontoxic up to 62.5 µg/mL and exhibited an IC_50_ of 112.46 µg/mL.

**Figure 3:**
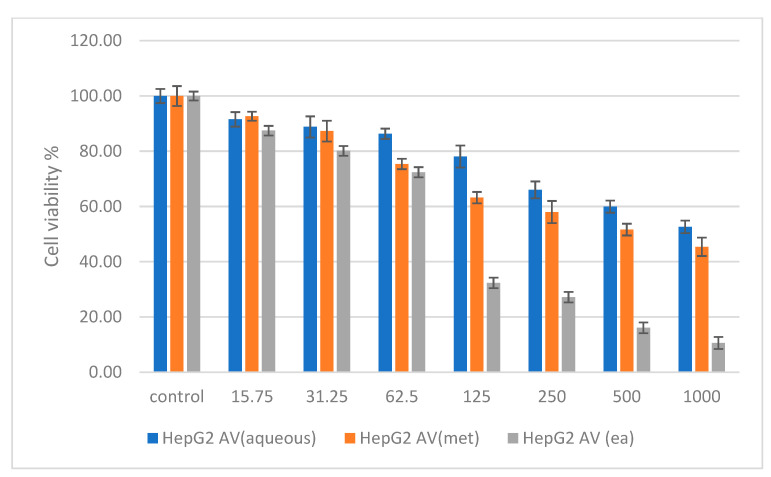
Cytotoxicity assay of *Artemisia verlotiorum* in aqueous, methanolic, and ethyl acetate extracts against human hepatocellular carcinoma HepG2 cells at 48 h.

**Figure 4:**
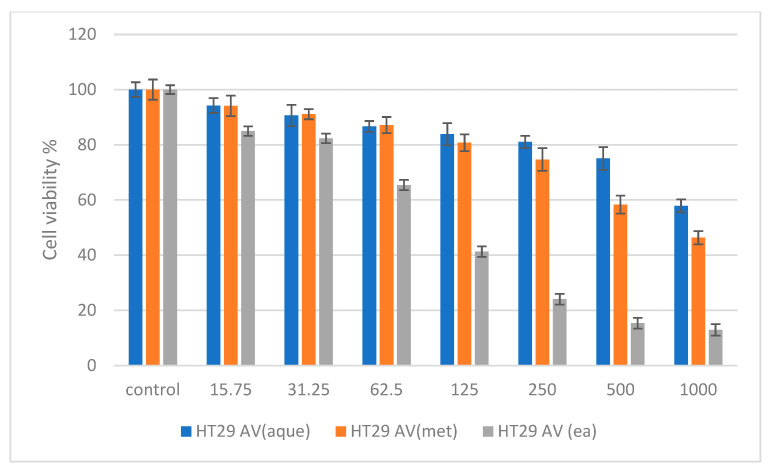
Cytotoxicity assay of *Artemisia verlotiorum* in aqueous, methanolic, and ethyl acetate extracts against human colorectal carcinoma HT29 cells at 48 h.

In Figure 3, HepG2 treated with *A. verlotiorum* Lamotte in aqueous extract exhibited non-toxic activity up to 250 µg/mL after 48 h of treatment, the methanolic extract was non-toxic up to 125 µg/mL and shows IC_50_ value of 722.39 µg/mL, and the ethyl acetate extract was non-toxic up to 62.5 µg/mL with an IC_50_ of 84.72 µg/mL. According to Figure 4, HT29 treated with *A. verlotiorum* in aqueous, methanolic, and ethyl acetate extract exhibited nontoxicity up to 500 µg/mL, 250 µg/mL, and 62.5 mg/mL (IC_50_ = 85.51 µg/mL), respectively. The IC_50_ value exhibited in the methanolic extraction of *A. verlotiorum* was 792.91 µg/mL. The ANOVA statistics revealed that no significant difference was found among the sample groups from 15.75 µg to 500 µg in NIH 3T3 cell concentrations. A significance difference of *p* ≤ 0.05 was exhibited by HepG2 (62.5–1000 µg/mL) cells and HT29 (250–1000 µg/mL) cells using ANOVA and Games–Howell multiple range test in all the cells that were tested. This shows that *A. verlotiorum* methanolic extraction is capable of inducing cell cytotoxicity in the human hepatocellular carcinoma without damaging normal cells.

## 3. Discussion

Despite being poorly studied, *Artemisia verlotorium* Lamotte is employed in traditional medicine in different countries such as Italy, Mauritius, and Brazil. Its infusion is administered as a remedy against hypertension in Italy [22]. In Mauritius, the plant decoction is recorded to be employed to treat fever, psoriasis, and influenza [23]. In Brazil, the inhabitants value the medicinal properties of this species against diseases related to circulatory, digestive, genito-urinary, and respiratory disorders [24]. To the best of our knowledge, this is the first report investigating the phytochemical and biological profile of *A. verlotiorum*.

To understand the biological activities that plant species exert, it is important to evaluate and explore their phytochemical constituents. The phytochemical evaluation in this study was performed by first evaluating the total phenolic, flavonoid, phenolic acid, and flavonol content of the *A. verlotorium* extracts. Based on the amount of extracted phytochemicals, it can be inferred that distilled water was the best solvent for extracting phenolics and phenolic acid from *A. verlotorium*. Methanol extracted the most flavonoid, while ethyl acetate was the best solvent for extracting flavonol.

The different proportions of distinct phytoconstituents displayed in the results section indicate different extraction behaviors of phenolics, flavonoids, phenolic acid, and flavonols in the different solvents. This can be explained by the fact that (i) other constituents that are not measured, such as lipids, oligosaccharides, sugars, and waxes, interfere with the extraction procedure and also demonstrate that (ii) different solvents have different abilities to penetrate the dried plant material [22]. Plant phenolic compounds are well-known antioxidants. With respect to human physiology, the consumption of foods high in phenolic compounds wards against the onset of chronic diseases such as cancers, cardiovascular diseases, and diabetes [23].

The phytoconstituents were characterized by HPLC. HPLC is a repeatable, vigorous, and versatile chromatographic method that can be employed to identify, quantify, and purify the distinct components in a crude plant extract [24]. For herbal fingerprinting, HPLC has gained notable popularity. It is a rapid analytical method with significant resolving power [25].

The compounds identified in the plant species through HPLC have notable and medicinal attributes, which are highlighted below. In endodontic therapy, citric acid is employed at a concentration of 50% to clean root canals post pulpectomy. This resulted in immaculate surfaces, as shown by scanning electron microscopy [26]. The use of 2–3% citric acid for wound care cleaned antibiotic-resistant *Pseudomonas aeruginosa* from the site [27]. Given its low cost, it is an economical antiseptic.

Rutin, also known as vitamin P or rutoside, is a non-toxic biflavonoid with a high safety margin, which can be produced at a low cost [28]. It has a broad spectrum of activity against several non-communicable ailments including diabetes, cancer, hypercholesteremia, and hypertension [29]. It possesses anti-allergic, anti-fungal, and antimicrobial properties [30]. The organoprotection benefits of rutin displayed in vitro and in vivo show that it can serve as an interesting compound for the management of long-term diabetes complications [31]. Rutin was injected safely into mice blood for the treatment of septic arthritis caused by *Candida albicans* [32]. In rats, rutin has also displayed anticonvulsant activity thought to be initiated via allosteric modulation of the GABA_A_ receptor complex via interaction at the benzodiazepine site [33].

Vicenin-2 is a secondary plant metabolite commonly found in many plant species such as *Ocimum sanctum*, *Artemisia capillaris*, and *Moringa oleifera,* among others. It is considered to be a safe and non-toxic flavonoid [34]. In vitro investigations conducted on this metabolite have shown that it holds promise pending further evaluation in diabetes mellitus, and colorectal, liver, and prostate cancer therapy. Indeed, it is a potent inhibitor of α-glucosidase, protein tyrosinase phosphatase 1B, and rat lens aldoctase reductase, and it limits the formation of advanced glycation products [20].

In colorectal cancer, vicenin-2 acted upon HT-29 cells to diminish the activities of T-cell factor/leukocyte erythroid factor and promote arrest of the HT-29 cell cycle at the G_2_M phase and was also found to induce apoptosis [35]. On the other hand, it displayed anti-angiogenic, apoptotic, and proliferative activity in prostate cancer cell lines. Its combined oral administration with the classical drug docetaxel in a mouse xenograft resulted in a synergistic association, leading to an increased efficacy in tackling androgen-independent prostate cancer [22,36]. In hepatocarcinoma cell lines, vicenin-2 inhibited STAT-3 phosphorylation and suppressed the activation of JAK [37].

Quinic acid is converted to tryptophan and nicotinamide in the gastrointestinal tract in situ, promoting DNA repair and inhibiting NF-kB [38]. A study by Cinkilic et al. demonstrated that quinic acid was a better protector of DNA from human lympophytes when exposed to radiation when compared with chlorogenic acid by decreasing damage from radiation by 5.99–53.7% versus 4.49–48.15% for chlorogenic acid [39]. The virucidal potential of quinic acids derivatives against all four dengue virus serotypes has been investigated in a human hepatoma cell line. Based on this study, their use has been deemed safe, and they could inhibit viral replication [40]. The dual use of quinic acid and undecanoic acid against the increasingly drug-resistant *Candida* sp. resulted in a synergistic activity among the two compounds, causing a decrease in biofilm production and several other virulence traits. Furthermore, this combination was found to be non-toxic and a promising avenue for researchers to explore to ward against *Candida* sp.-associated infections in medical implants [41].

Medioresinol has been deciphered as a novel PGC-1α activator, and in brain microvascular endothelial cells, it enhances the interaction of PGC-1α with PPARα, resulting in an escalation in GOT1 and PAH expressions, causing a reduction in the build-up of phenylalanine triggered by ischemia, depletion of mitochondrial ROS, and ultimately prophylaxis of pyroptosis of BMVECs, blood brain barrier disruption, and ischemic brain damage [42].

Eriodictyol is another flavonoid widely present in citrus fruits and vegetables [43]. It bears a plethora of pharmacological properties, including cancer and inflammation prevention and cardio, liver, diabetes protection. Under high-glucose conditions, it caused reuptake of glucose by human hepatocellular carcinoma cells and differentiated 3T3-L1 adipocytes in vitro [44]. The antioxidant and anti-inflammatory effects of eriodictyol in rats pretreated with the compound constricted acute lung injury. This was achieved due to eriodictyol’s ability to regulate Nrf2 and due to its inhibitory action on macrophages and cytokines [45].

Eriodictyol can target alpha-hemolysin, one of the virulence factors that increases the sensitivity of *Staphylococcus aureus* to host cells, which consequently leads to diverse other ailments [46]. In mice experimental models to determine neuroprotection, eriodictyol lead to the prophylaxis of neuronal death, smaller infarct area, as well as improved neurological and memory deficits caused by brain ischemia [47]. The chemical structures of some compounds from *A. verlotiorum* are shown in Figure 2.

Neochlorogenic acid (NCA) is a polyphenolic compound possessing formidable anti-bacterial, -oxidant, -pyretic, and -viral properties [48]. The anticancer activities of neochlorogenic acid (NCA) were investigated in vitro (on gastric cancer cells) and in vivo (nude mice xenograph model). In gastric cancer cells, the IC_50_ of NCA was 20 µM. Apoptosis in these cells was accompanied with loss in mitochondrial membrane potential as well as induced reactive oxygen species production within the cell. When the mice group treated with NCA was compared with the untreated group, a decrease in the volume and growth of tumors was noticed [49].

The aptitude of NCA for the management of inflammatory disorders was also scrutinized. Customarily, inflammatory disorders (IDs) stem from a surfeit of pro-inflammatory mediators, besides cytokines biosynthesized by macrophages. Anterior exposure to NCA narrowed the synthesis of IL-1β and IL-6, nitric oxide, prostaglandin E2, reactive oxygen species, tumor necrosis factor-α, by lipopolysaccharide-activated macrophages. In addition, the transcript and levels of nitric oxide synthase and cyclo-oxygenase 2 dwindled. Notably, NCA was highlighted as an AMPK/Nrf2 signal trigger, which can lead to a diminished macrophage reaction in acute and chronic IDs [50]. Tissue injury and microbial invasion is sensed by the microglia in the brain. Excessive inflammatory response by the microglia is a threat to neuronal cell survival and hence needs to be controlled.

The anti-inflammatory effects of NCA in this aspect was investigated in BV2 microglia. The suppression of synthesis of nitric oxide synthase, cyclooxygenase-2 expression, and nitric oxide formation ensued in a dose-dependent fashion in lipopolysaccharide activated BV2 microglia. Pro-inflammatory effects of interleukin -1 beta and tumor necrosis factor α were also reduced following a reduced concentration of these factors. These data demonstrate the positive influences that NCA can exert in ensuring neuroprotection [48]. The inflammatory responses in acute pneumonia were mitigated in vitro based on the results obtained by experimenting with NCA on LPS-stimulated inflammation in A549 cells. Several pro-inflammatory mediators were suppressed, including the activation of NF-kβ, and MAPKs signaling pathway phosphorylation was blocked [51]

Chlorogenic acid is essentially a notable dietary polyphenol possessing several pharmacological properties including antibacterial, antioxidant, antipyretic, antiobesity, anti-oxidant, antipyretic, antiviral, cardio- and hepato-protective, and neuroprotective benefits among several other health interests [52]. Inhibition of sortase A, an enzyme that plays an important role in the establishment of infections in multi-drug resistant *Staphylococcus aureus*, has become an important target for researchers.

In vitro, chlorogenic acid (CHA) inhibits sortase A (IC_50_ = 33.86 ± 5.55 μg/mL), along with the adhesion of *S. aureus* to fibrinogen. Chlorogenic acid exerts its inhibitory activity on sortase A by binding to the C184 and C192 sites in the enzyme. In mice, administration of CHA was beneficial in the prophylaxis of renal abscess [53]. The ability of CHA against cancer cells was investigated. It was found that CHA heals cancers by inducing differentiation among the cells in vitro and in vivo. In cancer cells that were exposed to CHA, the rate of proliferation, migration ability, and ATP production was reduced. The expression of SUMO1 in cancer cells was also increased; it initiated a cascade of events leading to the inhibition of lung cancer and hepatoma growth in tumor-affected mice, and the development of new tumors was blocked in young mice. In glioma cells, the effect of CHA was comparable to the conventional anticancer agent temozolamide, being present in both blood and brain post intraperitoneal administration, and was remarkably safe even at high doses [54].

The anti-inflammatory effect of CHA was studied in lipolysaccharide-stimulated RAW 264.7 cells. Intriguingly, CHA exerted an anti-inflammatory activity through a broad range of mechanisms which included lowered nitric oxide synthesis and a drop in the level of COX-2 and iNOS, without any cytotoxic effects. Other inflammatory markers were also inhibited, such as IL-1β, TNF-α, and IL-6, with increasing dose. A reduction in the expression level of Ninj1 and endotoxin-induced adhesion of macrophages was also noted, as well as attenuation of the nuclear translocation of NF-kβ [55].

The quest for the pursuit of novel antioxidant molecules is an ongoing practice by the scientific community. Antioxidants are well known for warding off a range of acute to chronic ailment conditions. This is the first study to report the phytoconstituents from the leaves of *A. verlotiorum*. Despite plants generally biosynthesizing a broad range of phytochemicals, the production of these molecules is largely dependent on the season of collection, the plant part from which extraction was performed, and the solvent employed for extraction [56].

The consumption of meat and meat products is deemed unhealthy due to their high content of saturated fats. Long-term consumption of meat and related products can lead to the onset of degenerative disorders. Substituting synthetic antioxidants in meat and meat with plant-derived antioxidants, such as vitamin A, C, and E, flavonoids, polyphenols, and terpenoids, could curb the incidence of a plethora of degenerative ailments, along with enhancing their shelf-life [57]. There are diverse methodologies that can be adopted for the determination of the antioxidant properties of plant extracts. Given that all the existent methods are accompanied by a range of advantages and drawbacks, it is wiser to perform various antioxidant evaluations of the extracts to obtain more conclusive results [58]. In this study, various antioxidant methods were employed, such as the DPPH, ABTS, FRAP, and CUPRAC methods.

The highest antioxidant potential was recorded for the aqueous extract, irrespective of the antioxidant assay performed. The aqueous extract had a higher proportion of metabolites, revealing the presence of several well-known antioxidant molecules such as rutin, vicenin-2, and eriodictyol. The antioxidant potential of the aqueous extract could also be related to the fact that out of the 25 phytochemicals identified in the different extracts employing the HPLC-ESI-MS^n^, the highest number of phytoconstituents (23) was identified from the aqueous extract, followed by the methanolic (21 phytoconstituents) and the ethyl acetate extract (14 phytoconstituents).

The main compounds are quantified in Table 3. The phenolic acid and flavonoids composition of the extracts were in the following order: aqueous extract > methanolic extract > ethyl acetate extract. The same trend was observed for the antioxidant activities. The highest composition in phenolic acids and flavonoid compounds may be directly correlated with the antioxidant capacities of the extracts. Phytoconstituents exert potent antioxidant activity whenever the flavonoids they biosynthesize consist of two or three hydroxyl components in the “B” ring of their skeleton [59,60]. Dicaffeoylglucaric acid was identified solely in the aqueous extract, which may explain the heightened antioxidant potential of the aqueous extract.

The results of this study allow us to suggest that *A. verlotiorum* leaves are a good natural source of antioxidants and can be substituted for commonly used synthetic antioxidants such as butylated hydroxy anisole and toluene [61].

Daily supplementation with antioxidants is considered important to ward off the dangerous effects of free radicals on the body. Free radicals such as reactive oxygen and nitrogen species are the product of normal cell metabolism; in general, they are atoms or molecules containing an odd number of electrons, making them highly reactive, unstable, and short lived, and they can exist independently. They react with other molecules, achieving stability and making them unstable, leading to a chain of events involved in the formation of other free radicals, which ultimately damage cells [62]. When present in low levels they exert useful immunological roles, such as warding off pathogenic microorganism [63]. Flavonoids are well recognized for their antioxidant properties as well as for their broad range pharmacological properties, which include antidiabetic, anti-inflammatory, antimicrobial, antimutagenic, anti-oestrogenic, anti-oxidant, antitumour, immunomodulatory, oestrogenic, and vaso-relaxant activities [64].

Nonetheless, when present in excess, they can alter the integrity of several important cellular biomolecules, such as DNA, lipids, and proteins, prompting an exaggerated oxidative stress in various human ailment conditions, such as in cardiovascular, respiratory, and neurodegenerative diseases, cataract, diabetes mellitus, and ageing, among others [65]. Hence, neutralizing free radicals with antioxidants is an interesting approach to handle the deleterious consequences of free radicals in aggravating several disease conditions [66].

The biological activities of *A. verlotiorum* extracts was further investigated regarding their potential against non-communicable chronic diseases such as diabetes mellitus, neurodegenerative diseases, and cancer. The activities of the plant species were investigated against the following enzymes: amylase, glucosidase, tyrosinase, glucosidase, acetylcholinesterase, and butyrylcholinesterase enzymes. All these enzymes are involved in the pathogenesis of diabetes mellitus and neurodegenerative diseases.

Tyrosinase is an enzyme involved in the process of melanogenesis instigating the biosynthesis of melanin [62]. Importantly, dysregulation in melanogenesis has an important implication in neurodegenerative diseases, such as Parkinson’s disease, as well as skin stains. Several cosmetic agents intended to remove skin stains and to reverse hyperpigmentation contain plant-based anti-tyrosinase active ingredients [67,68].

Furthermore, the formation of *o*-quinones following the action of tyrosinase causes the browning of plant-based foods and seafood. This impacts on their nutritional content, and given their physical states, they are less preferred by customers [69]. In this advent, it is interesting to find safe and effective tyrosinase compounds from the plant kingdom. Plant species with both anti-oxidant and anti-tyrosinase activities are striking in terms of their use as functional foods and applications in the cosmetics industry [56].

Cosmetics of natural origin are preferred by consumers in this modern era. Therefore, manufacturers are required to derive more of their ingredients that have skin rejuvenating properties from nature [70]. We investigated the anti-tyrosinase activities of the leaves of *A. verlotiorum*. The methanolic extract was the most potent inhibitor of the tyrosinase enzyme, followed by the ethylacetate and the aqueous extract. Dimethylated flavonoid and eriodictyol were the only metabolites that were commonly present in both the methanolic and ethyl acetate extract of *A. verlotiorum* leaves, which may have accounted for the heightened anti-tyrosinase activities of the extracts. Imen et al., 2015, investigated the effect of eriodictyol on melanogenesis using cultured murine melanoma cells (B16-F10). As the dose of eriodictyol increased, a more pronounced anti-tyrosinase activity was noted [71]. 

Neurodegenerative diseases are characterized by impaired cholinergic transmission. Inhibition of AChE and BChE is an important mechanism to slow down the depletion of acetylcholine in the brain and to restore to normal levels the neurotransmitter [72]. The AChE and BChE inhibitory effects of the ethyl acetate extract was most prominent compared to the methanolic extract and the aqueous extract, which was not active. This can be attributed to the fact that the ethyl acetate extract was richest in flavonol composition compared to the other two extracts.

Alzheimer’s disease (AD) is characterized by gradual memory and cognitive function loss. Inhibitors of the acetylcholinesterase and butyrylcholinesterasehave been found to increase the level of acetylcholine in the brain, hence maintaining better management of symptoms in Alzheimer’s disease patients. Several conventional AChE inhibitors are available on the market, such as donezepil, galanthamine, rivastigmine, and tacrine [73]. Nonetheless, they cannot reverse the disease’s progress and can only improve the symptoms associated with AD. In addition, they are related to several side effects. Since rivastigmine and galanthamine are structurally related to natural compounds, the focus is on research for such compounds that are inhibitors of cholinesterase enzymes and have fewer side effects [74].

Data from the World Health Organization demonstrate that diabetes figures have risen. Indeed, in 34 years, starting from the year 1980 to 2014, the available figures of people suffering from diabetes mellitus (DM) escalated from 108 to 422 million. DM directly leads to a vast range of other complications, such as loss of sight, failing kidneys, myocardial infarction, and lower limb amputation. In 2019 alone, 1.5 million deaths were attributed solely to DM [75].

Regarding the treatment and prophylaxis of DM, an important target, the α-glucosidase enzyme, has been recognized. In fact, its inhibition has been shown to control postprandial glycemia and control blood glucose levels in patients with DM. Conventional α-glucosidase inhibitors do exist, but their use is associated with certain side effects [76]. Hence, plants are invested to discover novel, safer, and cheaper α-glucosidase inhibitors.

Flavonols are the most widespread of the flavonoids [77]. An investigation by Yuan et al., 2014, demonstrated that α-amylase provides a binding site for flavonoids and that the binding occurs mainly through hydrophobic bonds [78]. Zhu et al. investigated the inhibitory effect of flavonoids on α-amylase and α-glucosidase enzymes. Indeed, the mechanisms through which these interactions take place was also elucidated, and it was concluded, firstly, that flavonoids specifically bind to amino acid residues in the active site of the enzyme, while excluding the binding of substrate, and secondly, upon interaction with the amino acid residues, the pathway to the active site is closed [79]. Hence, flavonoids can be considered as useful candidates that can be further explored for the management of diabetes mellitus.

Cancer causes one in every 6 deaths that occur worldwide and is the second leading cause of death, accounting for 9.6 million deaths in 2018. The most common forms of cancer in men are colorectal, lung, liver, prostate, and stomach cancer, while in women breast, cervical, colorectal, lung, and thyroid cancer are the most prominent forms. Cancer imposes an immeasurable emotional, financial, and physical burden on sufferers and their relatives. Around 300,000 new cases of cancers are detected among children between 0–19 years every year, and it is estimated that 30–50% of cancer cases can be prevented [80].

Chemotherapy is considered as one of the treatment alternatives among cancer patients. Several plants of African origin have demonstrated their effectiveness as derivatives of cytotoxic drugs that are employed in chemotherapy, such as *Taxus brevifolia* and *Catharanthus roseus*, which are the sources of paclitaxel and the vinca alkaloids, respectively [81].

Colon, rectum, and liver cancers were among the top killers for cancer in 2020. Other factors such as poor lifestyle and dietary habits lead to cancer. In addition, children are not spared from the deadly consequences of the disease worldwide. The goal in cancer therapy is early diagnosis and treatment (WHO, 2022). In addition, the drugs employed in chemotherapy are poorly selective for cancer cells, and their corresponding use is associated with a plethora of side/adverse effects [82]. Consequently, the reservoir of chemotherapeutic agents needs to be replenished with more selective and low-toxicity novel drugs.

*A. verlotiorum* methanolic extract is rich in phenolic, flavonoid, phenolic acid, and flavonols. Phenols have been recognized to exert cytotoxic activity as well as caspase-mediated apoptotic activity on various cancer cell lines. In addition, phenols also possess pro-oxidant activities, which further potentiates their antitumor properties [83].

Flavonoids exhibit their anticancer properties by acting through a number of mechanisms, including: (i) fine-tuning of enzymes involved in the scavenging of reactive oxygen species, (ii) aiding in cell cycle arrest, and (iii) promoting both autophagy and apoptosis, and down-turning the spread of invasiveness of cancer cells [84].

Neochlorogenic acid has been witnessed to exhibit antitumor activities in human gastric carcinoma cells in a study conducted by Fang et al., 2019. A recorded IC_50_ of 20 µM was recorded in this study. The mechanisms through which neochlorogenic acid acted were as follows: (i) induction of apoptosis through the loss of mitochondrial membrane potential combined with elevated intracellular reactive oxygen species production, and (ii) downregulation of important proteins of m-TOR/PI3/Akt signaling pathway. A six-week treatment in mice demonstrated that the tumor volumes in the treated group were less than in the untreated group [49].

In breast cancer, chlorogenic acid has been found to promote cellular apoptosis, inhibit metastasis, and boost antitumor immunity through the NF-kB signaling pathway [85]. In oral tumor cell lines, chlorogenic acid have shown to be cytotoxic, and it is suggested that it exerts this effect by acting through the hydrogen-peroxide-mediated oxidation mechanism [86].

In Hep-2 cells, dicaffeoquinic acids exhibited a median cytotoxic concentration greater than 1000 microM [87]. In leukemic cells, quercetin exerts a cytotoxic activity in a dose-dependent manner [88]. Several other studies have highlighted the cytotoxic potential of quercetin and the mechanisms through which it displays its effects. A study suggested that it may act as a prooxidant following its conversion to semiquinone and quinoidal product [89]. In breast cancer cells, human glioblastoma A172 cells quercetin exerts cytotoxic effects [88,90].

## 4. Materials and Methods

### 4.1. Plant Materials

*A. verlotiorum* was collected from the village of La Marie, Vacoas, Mauritius in 2019. The collected plant samples (whole plant) were deposited at the Mauritius Herbarium of the Mauritius Sugarcane Industry and Research Institute (MSIRI) situated in Réduit, Mauritius, for confirmation of their identity. Following identification, the plant was given the barcode number MAU0027519, and a voucher specimen of the species is available at the Mauritius Herbarium.

#### 4.1.1. Extraction of Phytochemicals

The leaves of *A. verlotiorum* were carefully cleaned with distilled water to free them from debris, after which they were kept in a well-ventilated area and protected against direct sunlight. Daily, the depletion in mass of the leaves was documented, and a steady mass was attained after 3 weeks. Then, the dried leaves were pulverized in a mechanical grinder. After pulverization, the leaves were macerated in a beaker by placing 100 g of each dried plant component distinctly in 1 L of (1) ethyl acetate and (2) methanol. Maceration of the plant components lasted for 14 days, during which the beakers were constantly shaken. Filtration then ensued with grade 1 Whatman^®^ filter paper.

A decoction was also formulated by placing 50 g of dried *A. verlotiorum* powder in 200 mL of distilled water and boiling the resultant mixture at 100 °C until it was reduced to one quarter of the original volume. The boiled mixture was then filtered through muslin cloth. Both the organic and the aqueous filtrates were then concentrated in a rotary evaporator at low temperature and pressure. The eventual crude extracts were then conserved at 4 °C in the dark for phytochemical screening and in vitro assays.

#### 4.1.2. Phytochemical Composition

The presence of the total bioactive compounds, including the total phenolic, flavonoid, phenolic acid, and flavonol content, was determined by colorimetric methods and expressed as mg of gallic acid, rutin, caffeic acid, and catechin per g of dried extract, respectively, as documented in previous studies [91,92].

#### 4.1.3. HPLC Analysis

The HPLC analysis was conducted using an Agilent Series 1100 with a G1315B diode array detector (Agilent Technologies, Santa Clara, CA, USA), a reversed-phase Luna Omega Polar C_18_ analytical column (150 × 3.0 mm; 5 µm particle size; Phenomenex, Torrance, CA, USA), and a Polar C_18_ Security Guard cartridge of 4 × 3.0 mm (Phenomenex). The mobile phases were as follows: water + formic acid 0.1% *v/v* (eluent A) and acetonitrile (eluent B). The gradient elution was as follows: 10–25% B in 0–25 min, 25% B in 25–30 min, 25–100% B in 30–35 min. Then, eluent B was returned to 10% with a 7 min stabilization time. The flow rate was 0.4 mL min^−1^.

The HPLC system was connected to an ion trap mass spectrometer (Esquire 6000, Bruker Daltonics, Billerica, MA, USA) equipped with an electrospray interface. The scan range was at *m*/*z* 100–1200 with a speed of 13,000 Da/s. The ESI conditions were as follows: drying gas (N_2_) flow rate and temperature, 10 L/min and 365 °C; nebulizer gas (N_2_) pressure, 50 psi; capillary voltage, 4500 V; capillary exit voltage, 117.3 V. The auto MS^n^ mode was used for the acquisition of MS^n^ data, with isolation width of 4.0 *m*/*z* and fragmentation amplitude of 0.6 V.

#### 4.1.4. Instrumentation for the HPLC Analysis

Chromatographic analyses were performed with an Agilent Series 1100 HPLC system with a G1315B diode array detector (Agilent Technologies) and an ion trap mass spectrometer (Esquire 6000, Bruker Daltonics) with an electrospray interface. Separation was performed in a Luna Omega Polar C_18_ analytical column (150 × 3.0 mm; 5 µm particle size) with a Polar C_18_ Security Guard cartridge (4 × 3.0 mm), both purchased from Phenomenex. Detailed chromatographic conditions are available in [93].

#### 4.1.5. Antioxidant and Enzyme Inhibitory Assays

Antioxidant assays were carried out according to previously reported methodologies [94]. The antioxidant potential was expressed as follows: mg Trolox equivalents (TE)/g extract in 2,2-diphenyl-1-picrylhydrazyl (DPPH) and 2,2’-azino-bis (3-ethylbenzothiazoline-6-sulfonic acid) (ABTS) radical scavenging, cupric reducing antioxidant capacity (CUPRAC) and ferric reducing antioxidant power (FRAP) tests, mmol TE/g extract in phosphomolybdenum assay (PDA), and mg ethylenediaminetetraacetic acid equivalents (EDTAE)/g extract in metal chelating assay (MCA).

The enzyme inhibitory assays were carried out according to previously reported methodologies [90]. The acetylcholinesterase (AChE) and butyrylcholinesterase (BChE) inhibition was expressed as mg galanthamine equivalents (GALAE)/g extract; tyrosinase inhibition was expressed as mg kojic acid equivalents KAE/g extract; and amylase and glucosidase inhibition were expressed as mmol acarbose equivalents (ACAE)/g extract.

#### 4.1.6. Cell Viability Assay

Cell viability assay was conducted to analyze the toxicity level of aqueous and methanolic extract to cell lines, which included normal human fibroblast (3T3), human hepatocellular carcinoma cells (HepG2), and human colorectal carcinoma cells (HT29), which were purchased from ATCC (Manassas, VI, USA). All the cells were grown using Roswell Park Memorial Institute (RPMI) 1640 medium (Nacalai Tesque, Kyoto, Japan) supplemented with 10% fetal bovine albumin (Sigma-Aldrich, St. Louis, MO, USA), and 1% antibiotics containing 10,000 units/mL penicillin and 10,000 μg/mL streptomycin (Nacalai Tesque, Kyoto, Japan). Cells were maintained and incubated in humidified 5% carbon dioxide, 95% room air, at 37 °C. Cells layers were harvested using 0.25% trypsin/1 mM-EDTA (Nacalai Tesque, Kyoto, Japan). This was followed by seeded in 96-well tissue culture plates at 1.0×104 cells/well for 24 h in an incubator to attach, and 80% confluence was attained for the treatment.

Methylthiazol tetrazolium (MTT)-based assay was carried out to determine the cell viability and cytotoxicity. Cells were treated with *A. verlotiorum* and in aqueous, methanolic, and ethyl acetate extracts where stock solutions were prepared by dissolving the compound in 1:1 of dimethyl sulfoxide (0.1%) and RPMI. Then, the mixture was further diluted in the same media to produce various final concentrations, ranging from 31.25 to 1000 μg/mL.

Once the cells were attached to the respective wells after 24 h, the tested compounds were added until a final volume of 100 μL well was obtained. After 48 h of incubation, 10 μL of MTT solution (5 mg/mL in PBS) was added in each well and further incubated for 3 h before being aspirated. Then, 100 μL of dimethyl sulfoxide was added per well in the dark and room temperature in order to dissolve the purple formazan salt. The intensity of the purple formazan solution, which reflects cell growth, was subsequently measured at a wavelength of 570 nm using a microplate reader (Biotek LE800, Winooski, VT, USA). All experiments were accomplished in triplicate, and the results are presented as the mean ± standard deviation.

#### 4.1.7. Cytotoxicity Studies on Normal (NIH 3T3) and Cancer (HepG2 and HT 29) Cell Lines

Cytotoxicity studies were conducted by treating with *A. verlotiorum* in aqueous, methanolic, and ethyl acetate extracts on normal fibroblast, 3T3 cells, and cancer (HepG2 and HT 29) cell lines. Various gradient concentrations of the samples were incubated for a maximum of 48 h.

#### 4.1.8. Statistical Analyses

Experimental results are presented as mean ± SD of triplicates. The differences between the experimental groups were investigated by one-way ANOVA, followed by Tukey’s test. Additionally, for the antioxidant assays, the differences were investigated among each different extract (aqueous, ethyl acetate, and methanolic). Further statistical analyses, such as among the distinct groups using Pearson correlation, were conducted in GraphPad Prism 9.0.2 software. Similarly, all the cytotoxicity assays were carried out in triplicate, and the standard deviations were calculated and are incorporated in the respective bar graphs. For the calculation of IC_50_, the *x*- against the *y*-axis was plotted, and we converted the *x*-axis values (concentration) to their log values, followed by nonlinear regression (curve fit) under the *xy* analysis to obtain a straight-line equation fit, *y* = *ax* + *b*, from which the regression line and then inhibition IC_50_ was calculated.

## 5. Conclusions

This study investigated the multi-faceted pharmacological activities of *A. verlotiorum* Lamotte extracts for the first time. Indeed, the aqueous extract had the highest total phenolic content, while the methanolic extract had the highest flavonoid composition. A total of 25 metabolites were detected from the extracts, with phenolic acids and flavonoids being the most preponderant metabolites. The antioxidant potential of the aqueous extract was noticeable and the best among all assayed extracts, irrespective of the methodology employed. The anti-amylase, glucosidase, tyrosinase, acetyl cholinesterase, and butyryl cholinesterase activities of the ethyl acetate extract were most prominent. Lastly, the cytotoxic potential of the methanolic extract was most remarkable given its cytotoxic potential on human hepatocellular carcinoma HepG2 and human colorectal carcinoma HT 29 cancer cell lines. Taken altogether, *A. verlotiorum* Lamotte is a noteworthy plant that can be further evaluated for its potential in the management and treatment of various chronic ailment conditions.

## 6. Recommendations

Given the long-standing use of *A. verlotiorum* in traditional systems of medicine in various regions of the world, it can be regarded as a safe antioxidant source. To further warrant this claim, more toxicology studies need to be conducted on this plant species. In addition, given that *Artemisia* species are well known for the presence of artemisin and other related diterpenoids, a study should be mounted to investigate how seasonal variations influence the biosynthesis of different phytochemicals such as artemisin within the plant species.

## Figures and Tables

**Table 1:** Bioactive composition of *A. verlotiorum* Lamotte extracts.

Samples	Total Phenolic Content (mgGAE/g)	Total Flavonoid Content (mgRE/g)	Total Phenolic Acid Content (mgCE/g)	Total Flavonol Content (mgCAE/g)
AV-Aq	104.42 ± 0.29 ^a^	10.70 ± 0.63 ^c^	59.26 ± 0.49 ^a^	1.63 ± 0.06 ^b^
AV-EA	25.28 ± 0.18 ^c^	13.81 ± 1.12 ^b^	na	2.23 ± 0.03 ^a^
AV-MEOH	56.50 ± 0.20 ^b^	31.38 ± 0.59 ^a^	32.38 ± 2.02 ^b^	1.14 ± 0.01 ^c^

Values are reported as mean ± SD of three parallel experiments. In the same column, the values marked with different letters (^a–c^) are significantly different at *p* < 0.05. AV-Aq: *A. verlotiorium* aqueous extract; AV-EA: *A. verlotiorium* ethyl acetate extract; AV-MEOH: *A. verlotiorium* methanolic extract; GAE: gallic acid equivalent; RE: rutin equivalent; CE: caffeic acid equivalent; CAE: catechin equivalent; na: not available.

**Table 2:** Characterization of the compounds found in the analyzed extracts of *Artemisia verlotiorum*.

No.	t*_R_* (min)	[M − H]^−^ *m*/*z*	*m*/*z* (% Base Peak)	Assigned Identification	MeOH	H_2_O	EA
1	1.8	191	MS^2^ [191]: 173 (46), 127 (14), 111 (100)	Citric acid *	✓	✓	
2	2.5	191	MS^2^ [191]: 173 (100)	Quinic acid		✓	
3	3.7	315	MS^2^ [315]: 153 (100) MS^3^ [315→153]: 123 (99), 109 (100), 108 (90)	Dihydroxybenzoic acid-O-hexoside	✓	✓	
4	5.2	353	MS^2^ [353]: 191 (100), 179 (23), 135 (7)	Neochlorogenic acid *	✓	✓	
5	5.7	371	MS^2^ [371]: 353 (16), 209 (100), 191 (38) MS^3^ [371→209]: 191 (100), 173 (5)	Caffeoylglucaric acid		✓	
6	8.9	353	MS^2^ [353]: 191 (100), 179 (5), 173 (5)	Chlorogenic acid *	✓	✓	✓
7	10.5	387	MS^2^ [387]: 369 (8), 207 (100), 163 (84) MS^3^ [387→207]: 163 (100)	Medioresinol	✓	✓	✓
8	11.3	533	MS^2^ [533]: 371 (100), 353 (10), 209 (24) MS^3^ [533→371]: 209 (100), 191 (34), 129 (6) MS^4^ [→→]:	Dicaffeoylglucaric acid		✓	
9	12.6	593	MS^2^ [593]: 503 (18), 473 (86), 383 (29), 353 (100), 325 (10)	Vicenin-2 *	✓	✓	
10	13.0	337	MS^2^ [337]: 191 (100) MS^3^ [337→191]: 127 (100)	5-p-Coumaroylquinic acid	✓	✓	✓
11	13.8	515	MS^2^ [515]: 353 (100), 191 (19), 179 (45) MS^3^ [515→353]: 191 (100), 179 (32), 173 (5), 135 (8)	Dicaffeoylquinic acid isomer	✓	✓	
12	14.5	367	MS^2^ [367]: 191 (100), 173 (5)	5-Feruloylquinic acid		✓	✓
13	15.3	463	MS^2^ [463]:417 (100), 255 (18), 161 (18) MS^3^ [463→417]: 255 (100), 161 (46)	Unknown	✓	✓	✓
14	19.6	609	MS^2^ [609]: 301 (100) MS^3^ [609→301]: 271 (35), 179 (100), 151 (76)	Rutin *	✓	✓	
15	20.7	463	MS^2^ [463]: 301 (100) MS^3^ [463→301]: 271 (52), 179 (100), 151 (74)	Quercetin-O-hexoside	✓	✓	
16	22.7	515	MS^2^ [515]: 353 (100), 191 (13), 179 (20), 173 (30) MS^3^ [515→353]: 191 (29), 179 (46), 173 (100), 135 (10)	Dicaffeoylquinic acid isomer	✓	✓	✓
17	24.1	515	MS^2^ [515]: 353 (100), 191 (18), 179 (4) MS^3^ [515→353]: 191 (100), 179 (33), 173 (3), 135 (5)	Dicaffeoylquinic acid isomer	✓	✓	✓
18	26.1	515	MS^2^ [515]: 353 (100), 191 (4), 179 (10), 173 (18) MS^3^ [515→353]: 191 (22), 179 (40), 173 (100), 135 (6)	Dicaffeoylquinic acid isomer	✓	✓	✓
19	27.0	549	MS^2^ [549]: 387 (100) MS^3^ [549→387]: 369 (5), 207 (100), 163 (58) MS^4^ [549→387→207]: 163 (100)	Medioresinol-O-hexoside	✓	✓	✓
20	27.9	625	MS^2^ [625]: 463 (100), 301 (46) MS^3^ [625→463]: 301 (100) MS^4^ [625→463→301]: 179 (100), 151 (76)	Quercetin-O-hexoside-O-hexoside	✓	✓	✓
21	29.7	529	MS^2^ [529]: 367 (100), 353 (33), 191 (31) MS^3^ [529→367]: 191 (100), 173 (4)	Feruloyl-caffeoyl-quinic acid	✓	✓	✓
22	30.4	515	MS^2^ [515]: 353 (100), 179 (12) MS^3^ [515→353]: 191 (100), 179 (76), 173 (29), 135 (15)	Dicaffeoylquinic acid isomer	✓	✓	
23	32.6	287	MS^2^ [287]: 269 (3), 151 (100), 135 (5), 125 (4)	Eriodictyol	✓		✓
24	35.3	593	MS^2^ [593]: 323 (92), 269 (100)	Unknown	✓	✓	✓
25	35.9	285	MS^2^ [285]: 285 (100), 241 (7)	Luteolin	✓	✓	✓
26	36.6	345	MS^2^ [345]: 330 (100) MS^3^ [345→330]: 315 (100) MS^4^ [345→330→315]: 287 (100)	Dimethylated flavonoid	✓		✓
27	37.1	677	MS^2^ [677]: 515 (100), 353 (22) MS^3^ [677→515]: 353 (100), 179 (5), 173 (18) MS^4^ [677→515→353]: 191 (100), 179 (60)	Tricaffeoylquinic acid	✓	✓	✓
28	38.2	204	MS^2^ [204]: 204 (100), 189 (24)	Unknown			✓
29	39.0	327	MS^2^ [327]: 291 (36), 229 (68), 211 (47), 171 (100)	Oxo-dihydroxy-octadecenoic acid	✓	✓	✓
30	40.4	329	MS^2^ [329]: 311 (32), 293 (41), 229 (86), 211 (100), 171 (70)	Trihydroxy-octadecenoic acid	✓	✓	✓
Total number of metabolites identified = 30					25	27	19

* Identified with analytical standards.

**Table 3:** Quantification of the main compounds detected in *Artemisia verlotiorum*.

N°	Assigned Identification	mg g^−1^ DE		
		MeOH	H_2_O	EA
*Phenolic acids*				
4	Neochlorogenic acid	0.88 ± 0.06 ^b^	3.5 ± 0.2 ^a^	---
6	Chlorogenic acid	6.8 ± 0.4 ^b^	18 ± 1 ^a^	---
11	Dicaffeoylquinic acid	0.45 ± 0.03 ^b^	3.6 ± 0.2 ^a^	---
16	Dicaffeoylquinic acid	5.3 ± 0.3 ^b^	15 ± 1 ^a^	0.14 ± 0.01 ^c^
17	Dicaffeoylquinic acid	44 ± 3 ^a^	45 ± 3 ^a^	2.8 ± 0.2 ^b^
18	Dicaffeoylquinic acid	19 ± 1 ^a^	20 ± 1 ^a^	0.71 ± 0.05 ^b^
**Total**		**76 ± 3** ^b^	**105 ± 4** ^a^	**3.7 ± 0.2** ^c^
*Flavonoids*				
20	Quercetin-O-Hex-O-Hex	0.16 ± 0.01 ^a^	0.13 ± 0.01 ^b^	---
**TIPC**		**76 ± 3** ^b^	**105 ± 4** ^a^	**3.7 ± 0.2** ^c^

Bold values represent the sum of each type of components. Means in the same line not sharing the same letter are significantly different at *p* < 0.05 probability level. Hex = hexoside.

**Table 4:** Relative peak areas and heat map of extracts of *Artemisia verlotiorum*.

Peak	Compound	MeOH	H_2_O	EA
1	Citric acid	2.72	2.19	0.00
2	Quinic acid	0.00	0.64	0.00
3	Dihydroxybenzoic acid-O-hexoside	0.12	0.26	0.00
4	Neochlorogenic acid	0.63	2.52	0.00
5	Caffeoylglucaric acid	0.00	0.70	0.00
6	Chlorogenic acid	6.47	7.61	0.96
7	Medioresinol	0.85	0.70	0.47
8	Dicaffeoylglucaric acid	0.00	0.85	0.00
9	Vicenin-2	0.38	0.97	0.00
10	5-p-Coumaroylquinic acid	0.60	0.53	0.13
11	Dicaffeoylquinic acid isomer	1.12	7.53	0.00
12	5-Feruloylquinic acid	0.00	0.98	0.40
13	Unknown	1.02	0.40	3.25
14	Rutin	0.62	0.82	0.00
15	Quercetin-O-hexoside	0.37	0.21	0.00
16	Dicaffeoylquinic acid isomer	10.12	19.28	1.21
17	Dicaffeoylquinic acid isomer	38.86	24.49	31.16
18	Dicaffeoylquinic acid isomer	26.19	23.40	7.24
19	Medioresinol-O-hexoside	1.81	2.05	0.00
20	Quercetin-O-hexoside-O-hexoside	1.31	0.52	0.17
21	Feruloyl-caffeoyl-quinic acid	1.02	0.64	6.30
22	Dicaffeoylquinic acid isomer	0.52	0.05	0.00
23	Eriodyctiol	0.74	0.00	13.49
24	Unknown	0.11	0.14	2.44
25	Luteolin	0.07	1.29	4.42
26	Dimethylated flavonoid	0.40	0.00	6.28
27	Tricaffeoylquinic acid	1.23	0.30	0.13
28	Unknown	0.00	0.00	4.36
29	Oxo-dihydroxy-octadecenoic acid	0.96	0.34	5.99
30	Trihydroxy-octadecenoic acid	1.78	0.60	11.61

**Table 5:** Antioxidant activities of *A. verlotiorum*.

Samples	DPPH (mgTE/g)	ABTS (mgTE/g)	FRAP (mgTE/g)	CUPRAC (mgTE/g)	Metal Chelating (mgEDTAE/g)	Phosphomolybdenum (mmolTE/g)
AV-Aq	370.26 ± 0.89 ^a^	669.46 ± 10.48 ^a^	565.77 ± 3.57 ^a^	875.90 ± 17.72 ^a^	21.39 ± 0.84 ^a^	3.39 ± 0.12 ^a^
AV-EA	33.46 ± 1.71 ^c^	85.11 ± 1.92 ^c^	87.33 ± 3.66 ^c^	177.60 ± 0.69 ^c^	18.87 ± 1.63 ^a,b^	2.91 ± 0.17 ^b,c^
AV-MEOH	192.59 ± 0.58 ^b^	307.93 ± 4.82 ^b^	243.60 ± 0.68 ^b^	462.67 ± 15.18 ^b^	12.01 ± 0.89 ^c^	2.69 ± 0.17 ^c^

Values are expressed as mean ± SD of three replicates. In the same column, the values affected with different letter (^a–c^) are significantly different at *p* < 0.05. AV-Aq: *A. verlotiorium* aqueous extract; AV-EA: *A. verlotiorium* ethyl acetate extract; AV-MEOH: *A. verlotiorium* methanolic extract. Values are reported as mean ± SD of three parallel experiments. TE: Trolox equivalent; EDTAE: EDTA equivalent.

**Table 6:** Results of the tyrosinase, cholinesterase, amylase, and glucosidase assays.

Samples	AChE Inhibition (mgGALAE/g)	BChE Inhibition (mgGALAE/g)	Tyrosinase Inhibition (mgKAE/g)	Amylase Inhibition (mmolACAE/g)	Glucosidase Inhibition (mmolACAE/g)
AV-Aq	Na ^c^	Na ^c^	53.26 ± 1.61 ^c^	0.09 ± 0.01 ^a^	0.31 ± 0.02 ^c^
AV-EA	5.48 ± 0.09 ^a^	6.83 ± 0.72 ^b^	122.14 ± 2.36 ^b^	0.68 ± 0.04 ^b,c^	1.71 ± 0.01 ^a^
AV-MEOH	3.28 ± 0.10 ^b^	2.93 ± 0.13 ^b^	126.99 ± 1.71 ^a^	0.63 ± 0.03 ^c^	0.49 ± 0.09 ^b^

Values are expressed as mean ± SD of three replicates. In the same column, the values affected with different letter (^a–c^) are significantly different at *p* < 0.05. AV-Aq: *A. verlotiorium* aqueous extract; AV-EA: *A. verlotiorium* ethyl acetate extract; AV-MEOH: *A. verlotiorium* methanolic extract; GALAE: galatamine equivalent; KAE: kojic acid equivalent; ACAE: acarbose equivalent. na: not active.

## Data Availability

Not applicable.

## Sample Availability

Samples of the compounds are available from the authors.
